# High dose tigecycline in critically ill patients with severe infections due to multidrug-resistant bacteria

**DOI:** 10.1186/cc13858

**Published:** 2014-05-05

**Authors:** Gennaro De Pascale, Luca Montini, Mariano Alberto Pennisi, Valentina Bernini, Riccardo Maviglia, Giuseppe Bello, Teresa Spanu, Mario Tumbarello, Massimo Antonelli

**Affiliations:** 1 Department of Intensive Care and Anesthesiology, Catholic University of the Sacred Heart, Agostino Gemelli Hospital, Largo A. Gemelli, 800168 Rome, Italy; 2 Institute of Infectious Diseases, Catholic University of the Sacred Heart, Agostino Gemelli Hospital, Rome, Italy; 3 Institute of Microbiology, Catholic University of the Sacred Heart, Agostino Gemelli Hospital, Rome, Italy

## Abstract

**Introduction:**

The high incidence of multidrug-resistant (MDR) bacteria among patients admitted to ICUs has determined an increase of tigecycline (TGC) use for the treatment of severe infections. Many concerns have been raised about the efficacy of this molecule and increased dosages have been proposed. Our purpose is to investigate TGC safety and efficacy at higher than standard doses.

**Methods:**

We conducted a retrospective study of prospectively collected data in the ICU of a teaching hospital in Rome. Data from all patients treated with TGC for a microbiologically confirmed infection were analyzed. The safety profile and efficacy of high dosing regimen use were investigated.

**Results:**

Over the study period, 54 patients (pts) received TGC at a standard dose (SD group: 50 mg every 12 hours) and 46 at a high dose (HD group: 100 mg every 12 hours). Carbapenem-resistant *Acinetobacter.baumannii* (bla_OXA-58_ and bla_OXA-23_ genes) and *Klebsiella pneumoniae* (bla_KPC-3_ gene) were the main isolated pathogens (n = 79). There were no patients requiring TGC discontinuation or dose reduction because of adverse events. In the ventilation-associated pneumonia population (VAP) subgroup (63 patients: 30 received SD and 33 HD), the only independent predictor of clinical cure was the use of high tigecycline dose (odds ratio (OR) 6.25; 95% confidence interval (CI) 1.59 to 24.57; *P* = 0.009) whilst initial inadequate antimicrobial treatment (IIAT) (OR 0.18; 95% CI 0.05 to 0.68; *P* = 0.01) and higher Sequential Organ Failure Assessment (SOFA) score (OR 0.66; 95% CI 0.51 to 0.87; *P* = 0.003) were independently associated with clinical failure.

**Conclusions:**

TGC was well tolerated at a higher than standard dose in a cohort of critically ill patients with severe infections. In the VAP subgroup the high-dose regimen was associated with better outcomes than conventional administration due to Gram-negative MDR bacteria.

## Introduction

Tigecycline (TGC) is the first glycylcycline of the tetracycline antibiotic class approved in Europe for the treatment of complicated skin and skin-structures infections (cSSI), complicated intra-abdominal infections (cIAI), at a dose of 50 mg twice daily after a 100 mg loading dose, [1].

*In vitro* this antibiotic has shown good antibacterial activity against most of aerobic and anaerobic bacteria, including multidrug-resistant (MDR) Gram-negative bacteria. However *Pseudomonas aeruginosa*, *Proteus spp.* and *Providencia spp.* are intrinsically resistant [1].

A recent Food and Drug Administration (FDA) alert [2] announced an increased TGC-attributable mortality, thus discouraging its adoption for severe nosocomial infections. However, due to the scarcity of other effective antimicrobials its use is frequently extended to the treatment of colistin-resistant bacteria [3].

Because the area under the plasma concentration versus time (AUC) to microorganism minimal inhibitory concentration (MIC) ratio (AUC/MIC ratio) is the major determinant of antimicrobial activity of TGC, some authors have proposed increased daily dosages for treating severe infections due to MDR bacteria [4]. Clinical experience with doses >100 mg daily is very limited, but data reported to date suggest that TGC may be useful and well-tolerated at higher doses [5,6].

Thus we performed a retrospective analysis of prospectively collected data from critically ill patients who received TGC for microbiologically confirmed severe infections, to investigate its efficacy and safety at higher than standard doses.

## Methods

### Study site, subjects and design

The study was conducted in the 18-bed adult ICU of a tertiary university teaching hospital admitting approximately 900 patients per year. This study was approved by our Ethical Committee (Catholic University’s Ethics Committee (approval number:14599/13)) that waived the need for informed consent, due to its retrospective design. All patients consecutively admitted to our ICU between 1 June 2009 through 31 May 2012 who received TGC for a microbiologically documented infection were evaluated. TGC treatment should last at least three days including the loading dose (LD). Data were extracted from patients’ medical records and computerized hospital databases according to a pre-defined questionnaire. These data included demographic characteristics, medical history, clinical and laboratory findings, the simplified acute physiology score II (SAPS II) and sequential organ failure assessment (SOFA) score, the occurrence of abnormal laboratory measures, type of treatment and outcome. The main outcomes of patients were evaluated according to TGC dosages they received, and type of infections, separately analyzing the subgroup of patients with ventilator-associated pneumonia (VAP).

### Definitions

Patients who were treated with TGC 50 mg every 12 hours after a 100-mg LD were defined as the standard dose group (SD). Those ones who received 100 mg every 12 hours after a 200 mg LD were classified as the high dose group (HD).

The diagnosis of VAP was established when a new, persistent, progressive radiographic lung infiltrate was present ≥48 hours following tracheal intubation and when two or more of the following clinical criteria were met: (1) new onset of purulent bronchial secretions; (2) body temperature >38.8°C or <35.5°C; and (3) white blood cell count >10,000/mm^3^ or <4,000/mm^3^[7]. All episodes were microbiologically confirmed by quantitative cultures of bronchoalveolar lavage (≥10^4^ cfu/ml). The diagnosis of IAI and cSSTIs and bloodstream infections (BSIs) were made according to current guidelines [8-10].

Infection onset coincided with the collection date of the first microbiological sample culture yielding the study isolate (index culture). Septic shock was defined as recommended by the American College of Chest Physicians/Society of Critical Care Medicine Consensus Conference Committee [11]. Safety and adverse events (AE) were determined through the biochemical abnormalities documented in medical records according to the Department of Health and Human Services - Common Terminology Criteria for Adverse Events (DHHS-CTCAE v.3.0) classification [12]. The severity of AE was graded from 1 to 5 [12].

Clinical cure was defined as the complete resolution of all signs and symptoms of the infection by the end of TGC therapy. Improvement or lack of progression of all abnormalities on chest radiographs was also required for VAP [13]. Microbiological eradication was defined as the absence of the original pathogens from the culture of the specimens subsequently collected from the original site. Clinical outcomes were independently evaluated by two physicians (GDP, VB) who were blinded to the treatment. When judgments were discordant (about 5% of patients), the reviewers reassessed the data and reached a consensus decision.

The initial antimicrobial regimen (that is, that used before *in vitro* susceptibility data were available for the isolated bacteria) was classified as inadequate (IIAT) when it did not include any agent displaying *in vitro* activity against the isolated pathogen/pathogens.

### Microbiology analysis

Strains were identified to the species level with the matrix-assisted laser desorption ionization-time-of-flight (MALDI-TOF) mass spectrometry (MS) (BrukerDaltonik). The antibiotic susceptibility profiling of isolates had been performed with the Vitek 2 system (bioMérieux, Marcy l'Etoile, France). The Clinical and Laboratory Standards Institute (CLSI) criteria were used to interpret the results [14]. TGC minimum inhibitory concentrations (MICs) were identified with the Sensititre broth microdilution method (Trek Diagnostic Systems, Cleveland, OH, USA); isolates were considered susceptible if the MIC was ≤2 mg/L and resistant if the MIC was ≥8 mg/L [15]. Multidrug-resistance was defined as acquired non-susceptibility to at least one agent in three or more antimicrobial categories, extensive drug-resistance (XDR) was defined as non-susceptibility to at least one agent in all but two or fewer antimicrobial categories and pandrug-resistance (PDR) was defined as non-susceptibility to all agents in all antimicrobial categories [16]. The presence of blagenes conferring resistance to carbapenems was determined by polymerase chain reaction (PCR) and sequencing, as previously described [17,18].

### Statistical analysis

The Kolmogorov-Smirnov test was used to value the variables distribution. The data with a non-normal distribution were assessed with Mann–Whitney test and the median and selected centiles (25th to 75th) value was given. The data with a normal distribution were assessed with the Student *t*-test. Categorical variables are presented as proportions and were analyzed with the use of the chi-square test or Fisher exact test, as appropriate. A *P*-value <0.05 was considered significant. The crude odds ratio (OR) and 95% CI were calculated for each variable. We included all variables in the multivariable logistic regression if they achieved a *P*-value of less than or equal to 0.2 at the univariate analysis. A stepwise selection procedure was used to select variables for inclusion in the final model. The Hosmer-Lemeshow goodness-of-fit test and the receiver operating characteristic (ROC) curve analysis were used to assess the goodness of the logistic final model. All statistical analyses were performed using the Intercooled Stata program, version 11, for Windows (Stata Corporation, College Station, TX, USA).

## Results

### Incidence and patient characteristics in the overall population

During the study period, out of 2,120 patients admitted to our ICU, 134 received TGC with an incidence of 63.2 treated patients per 1,000 ICU admissions. Only 100 patients fulfilled the inclusion criteria and were considered for the retrospective analysis: 63 (63%) were affected by VAP and 37 by other infections: IAI (n = 23, 23%), primary BSI (n = 7, 7%) and cSSTI (n = 7, 7%).

**Figure 1:**
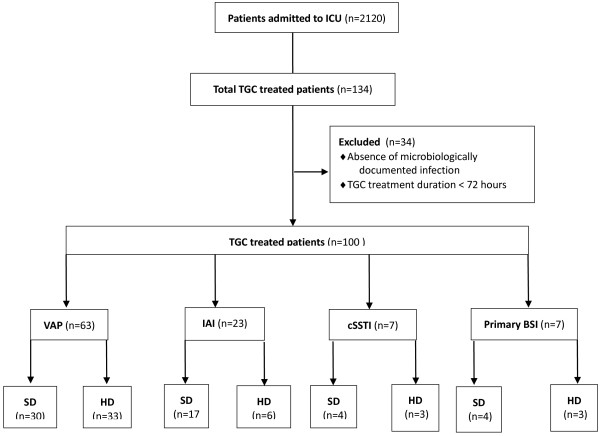
**Flow chart of study inclusion process.** TGC, tigecycline; SD, standard dose; HD, high dose; VAP, ventilator-associated pneumonia; IAI, intra-abdominal infection; cSSTI, complicated skin and soft tissue infection; BSI, bloosdstream infection.

Fifty-four patients received the SD of TGC and 46 the HD. In the VAP subgroup 47.6% (30/63) received SD and 52.4% (33/63) HD. The flowchart of the study is shown in Figure 1. Ninety percent (n = 109) of the 121 causative organisms were Gram-negative, most often *Klebsiella pneumoniae* (n = 50) and *Acinetobacter baumannii* (n = 34). Carbapenem resistance was detected in all *A. baumannii* isolates, and in all but five (three VAP, two cSSTI) *K. pneumoniae* isolates. PCR and sequencing analysis revealed that all *A. baumannii* isolates carried *bla*_OXA-58_ or bla_OXA-23_ genes while the 45 carbapenem-resistant strains of *K. pneumoniae* contained *bla*_KPC-3._

Of the remaining 37 bacteria, 20 (*Escerichia coli* (n = 8); *Enterobacter spp*. (n = 6); *Serratia marcescens* (n = 4); and *Stenotrophomonas maltophilia* (2)) were isolated in VAP patients and 17 in the remaining patients (*Escerichia coli* (n = 2); *Enterobacter spp*. (n = 1); *Morganella morganii* (n = 1); *Citrobacter freundii* (n = 1); *Staphylococcus aureus* (n = 7); *Staphylococcus epidermidis* (n = 2); and *Enterococcus spp*. (n = 3)). Of these microorganisms 22 (59.5%) were classified as MDR and most of them were isolated in patients treated with SD TGC (48% versus 20%, *P* <0.01).

**Figure 2:**
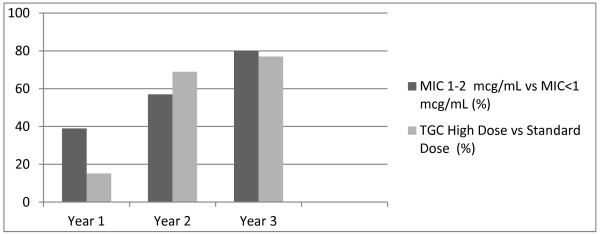
Correlation between minimum inhibitory concentration (MIC) values and standard-dose (SD) tigecycline (TGC) use during the three-year study.

Infections due to less susceptible bacteria (TGC minimal inhibitory concentration (MIC) value 1 to 2 mcg/mL) were mainly treated with higher doses (68% versus 36%, *P* <0.01). During the three-year study the percentage of patients treated with HD TGC increased (15% (year 1) versus 77% (year 3); *P* = 0.01) according to a significant increase of bacteria harboring MIC values of 1 to 2 mcg/mL (39% (year 1) versus 80% (year 3); *P* = 0.03) (Figure 2).

### Outcomes of patients with VAP according to dose of TGC

Patients with VAP treated with SD or HD TGC were similar in their baseline clinical conditions, disease severity and principal comorbidities (Table 1). Although not statistically significant, duration of TGC therapy was longer (9.0 versus 6.5 days, *P* = 0.13) in the HD group than in the SD one. The rate of IIAT (57.5% versus 46.6%, *P* = 0.38) was similar in the two groups. HD TGC was preferred for treating infections caused by difficult-to-treat bacteria (TGC MIC 1 to 2 mcg/mL) and to *K. pneumoniae* (*P* <0.001; *P* = 0.03).

**Table 1:** Clinical characteristics of the 63 patients with VAP in the standard-dose (SD) and high-dose (HD) tigecycline (TGC) groups

**Variable**	**SD TGC group (n = 30)**	**HD TGC group (n = 33)**	***P*-value**
Age, years, mean ± standard deviation	64.5 ± 16.9	60.7 ± 12.5	0.31
Male, n (%)	17 (56.6)	24 (72.7)	0.18
SAPS II score, mean ± standard deviation	51.3 ± 14.4	48.5 ± 14.9	0.46
SOFA score at infection occurrence, mean ± standard deviation	7.8 ± 3.2	7.4 ± 2.7	0.49
Septic shock at infection occurrence, n (%)	10 (33.3)	18 (54.5)	0.09
ARDS at infection occurrence, n (%)	2 (6.6)	7 (21.2)	0.09
**Diagnosis on ICU admission, n (%)**			
Medical	24 (80)	24 (72.7)	0.49
Non-medical	6 (20)^b^	9 (27.3)^c^	
**Comorbidities, n (%)**			
CHF	12 (40)	15 (45.4)	0.66
COPD	4 (13.3)	6 (18.1)	0.59
CRF	4 (13.3)	5 (15.1)	0.83
Malignancies	3 (10)	7 (21.2)	0.22
Diabetes	5 (16.6)	8 (24.2)	0.45
Immunosuppressive status	3 (10)	6 (18.1)	0.35
Comorbidities >1	17 (57)	21 (63.6)	0.57
**Microbiological and therapeutically aspects**			
Concomitant use of other active antibiotics, n (%)	24 (80)	29 (87.9)	0.39
Duration of TGC treatment, days, median (IQR)	6.5 (4 to 12)	9 (6 to 12)	0.13
Initial inadequate treatment, n (%)	14 (46.6)	19 (57.5)	0.38
**Responsible pathogens, n (%)**			
*Acinetobacter baumannii* XDR	13 (43.3)	15 (45.4)	0.86
*Klebsiella pneumoniae* MDR/XDR	10 (33.3)	20 (60.6)	0.03
Other bacteria	14 (46.6)	6 (18.1)	0.01
MIC value 1 to 2 mcg/mL^a^	8 (32)	23 (79.3)	<0.01
**Clinical and microbiological outcome, n (%)**			
ICU mortality	20 (66.6)	16 (48.4)	0.14
Clinical cure	10 (33.3)	19 (57.5)	0.05
Microbiological eradication	7 (30.4)	12 (57.1)	0.07

^a^MIC value was analyzed in 56 patients (26 in the SD TGC group and 30 in the HD TGC group); ^b^two surgical and four trauma; ^c^six surgical and four trauma. SAPS II, simplified acute physiology score II; SOFA, sequential organ failure assessment; ARDS, acute respiratory distress syndrome; CHF, chronic heart failure; COPD, chronic obstructive pulmonary disease; CRF, chronic renal failure; MDR, multidrug resistant; XDR, extensively drug-resistant; MIC, minimum inhibitory concentration.

Of the 63 patients with VAP, 53 (84.1%) were treated concomitantly with other active antibiotics, without differences between the two groups (87.9% in the HD versus 80% in the SD group; *P* = 0.39). Colistin was used in 35 cases (6,000,000 to 9,000,000 IU/day divided into two to three daily doses, after the loading dose), gentamycin in 12 cases (5 to 7 mg/kg q 24 h) and amikacin in 6 cases (15 to 20 mg q 24 h). MICs of colistin, gentamicin and amikacin were of < = 1 mg/L, <= 2 mg/L, and < = 2 mg/L, respectively. All dosages were adjusted for creatinine clearance if necessary. Gentamycin and amikacin peak and trough plasmatic levels were routinely checked.

The clinical cure rate and microbiological eradication percentage were higher when TGC was used at higher doses (57.5 versus 33.3; *P* = 0.05, and 57.1% versus 30.4%; *P* = 0.07, respectively). However, microbiological eradication was analyzed in only 44 patients, 23 in the SD TGC group and 21 in HD TGC group. The overall mortality in the VAP group was 57%, without differences between the two groups.

### Predictors of clinical cure in patients with VAP

The univariate analysis (Table 2) of the 63 patients with VAP showed that individuals with clinical failure were older, had a higher SOFA score and a shorter duration of TGC treatment than the patients with a successful clinical outcome. No specific antibiotic combination was associated with a better outcome. The logistic regression analysis indicates that the use of high dose TGC was the sole independent predictor of clinical cure (OR 6.25, 95% CI 1.59, 24.57), instead a higher SOFA score (OR 0.66, 95% CI 0.51, 0.87), and IIAT (OR 0.18, 95% CI 0.05, 0.68) was significantly associated with clinical failure (Table 3).

**Table 2:** Univariate analysis of factors associated with clinical cure in 63 patients with VAP

**Variable**	**Clinical cure (n = 29)**	**Clinical failure (n = 34)**	***P*-value**	**Odds ratio**	**95% CI**
Age, years, mean ± standard deviation	58.5 ± 16.9	66.1 ± 11.8	0.04	-	-
Male, n (%)	23 (79.3)	18 (52.9)	0.02	3.4	0.98, 12.67
SAPS II score, mean ± standard deviation	48.2 ± 13.7	51.2 ± 15.4	0.42	-	-
SOFA score at infection occurrence, mean ± standard deviation	6.4 ± 2.4	8.6 ± 3.0	0.003	-	-
**Causes of ICU admission, n (%)**					
Medical	19 (65.5)	29 (85.2)	0.06	0.32	0.07, 1.27
Non-medical	10 (34.4)^**a**^	5 (14.7)^**b**^		-	-
**Comorbidities**					
CHF, n (%)	13 (44.8)	14 (41.1)	0.77	1.16	0.37, 3.54
COPD, n (%)	6 (20.6)	4 (11.7)	0.33	1.95	0.4, 10.47
CRF, n (%)	4 (13.7)	5 (14.7)	0.91	0.92	0.16, 4.85
Malignancies, n (%)	4 (13.7)	6 (17.6)	0.74	0.75	0.14, 3.59
Diabetes, n (%)	6 (20.6)	7 (20.5)	0.99	1	0.24, 4.07
Immunosuppressive status, n (%)	4 (13.7)	5 (14.7)	0.91	0.92	0.16, 4.84
Comorbidities >1, n (%)	17 (58.6)	21 (61.7)	0.79	0.87	0.28, 2.72
Initial inadequate treatment, n (%)	11 (37.9)	22 (64.7)	0.03	0.33	0.1, 1.04
Duration of TGC treatment, days, median (IQR)	11 (6 to 13)	7 (3 to 10)	0.03	-	-
Septic shock at infection occurrence, n (%)	11 (37.9)	17 (50)	0.33	0.61	0.2, 1.87
Standard-dose group, n (%)	10 (34.4)	20 (58.8)	0.05	-	-
High-dose group, n (%)	19 (65.5)	14 (41.1)		2.71	0.86, 8.64
Concomitant use of other active antibiotics, n (%)	26 (89.7)	27 (79.4)	0.27	2.24	0.44, 14.72
ICU LoS, days, median (IQR)	35 (16 to 61)	26 (14 to 33)	0.06	-	-
ICU LoS before infection occurrence, days, median (IQR)	17 (6 to 27)	10.5 (4 to 21)	0.14	-	-
Duration of MV, days, median (IQR)	21 (13 to 43)	23 (11 to 33)	0.83	-	-
Duration of MV before infection occurrence, days, median (IQR)	17 (5 to 22)	11 (5 to 18)	0.21	-	-
**Responsible pathogens, n (%)**					
*Acinetobacter baumannii* XDR	13 (44.8)	15 (44.1)	0.95	1.02	0.33, 3.13
*Klebsiella pneumoniae* MDR/XDR	16 (55.1)	14 (41.1)	0.26	1.75	0.58, 5.38

Non-medical causes included: ^a^six surgical and five trauma; ^b^two surgical and three trauma. Data are shown as median with 25th and 75th percentile, until otherwise indicated. VAP, ventilator-associated pneumonia; SAPS II, simplified acute physiology score II; SOFA, sequential organ failure assessment; CHF, chronic heart failure; COPD, chronic obstructive pulmonary disease; CRF, chronic renal failure; TGC, tigecycline; LoS, length of stay; MV, mechanical ventilation; MDR, multidrug resistant; XDR, extensively drug-resistant. ‘-’ stands for Not Applicable.

**Table 3:** Logistic regression analysis of factors associated with clinical cure in 63 patients with ventilator-associated pneumonia

**Variable**	**Multivariate analysis**		
	**Odds ratio**	**95% CI**	***P-*** **value**
SOFA score at infection occurrence	0.66	0.51, 0.87	0.003
Initial inadequate treatment	0.18	0.05, 0.68	0.01
High-dose tigecycline group	6.25	1.59, 24.57	0.009

SOFA, sequential organ failure assessment.

### Adverse events

In the overall population the rate of abnormal laboratory measures during the treatment period was similar between SD and HD TGC-treated patients. No patients required TGC discontinuation or dose reduction. For all AE the maximum grade was 2 (moderate). Similar results were also obtained on stratifying patients by the type of infection (that is, VAP versus infections other than VAP) (Table 4).

**Table 4:** Comparison of adverse events in the SD TGC group and HD TGC group

**Abnormal laboratory measures (overall population)**	**Total population (n = 100)**	**SD TGC group (n = 54)**	**HD TGC group (n = 46)**	***P*-value**
BUN increase, n (%)	13 (13)	5 (9)	8 (17)	0.25
Impaired renal function, n (%)	19 (19)	11 (20)	8 (17)	0.8
Impaired hepatopancreatic function,n (%)	18 (18)	9 (17)	9 (19.5)	0.9
Impaired hematological function, n (%)	9 (9)	6 (11)	3 (6.5)	0.5
**Abnormal laboratory measures (VAP subgroup)**	**Total population (n = 63)**	**SD TGC group (n = 30)**	**HD TGC group (n = 33)**	***P*-value**
BUN increase, n (%)	8 (13)	3 (10)	5 (15)	0.7
Impaired renal function, n (%)	12 (19)	6 (20)	6 (18)	1
Impaired hepatopancreatic function, n (%)	11 (17.5)	4 (13)	7 (21)	0.6
Impaired hematological function, n (%)	4 (6)	1 (3)	3 (9)	0.6

Values are presented for all 100 patients with ventilator-associated pneumonia (VAP) and other VAP infections. All adverse events were graded 1 to 2. TGC treatment was interrupted in any patient with suspected severe adverse events.

TGC, tigecycline; SD standard dose; HD, high dose; BUN, blood urea nitrogen.

## Discussion

In this study we found that the use of HD TGC (100 mg every 12 hours following a 200 mg loading dose) was well-tolerated in a cohort of critically ill patients affected by nosocomial infections. In the VAP subgroup population (all episodes due to Gram-negative germs), the HD TGC was independently associated with a six-fold increase in clinical cure. Conversely an IIAT and a higher SOFA score were independent predictors of clinical failure. The association of increased TGC dosage and improved outcome is coherent with previous clinical observations regarding this and other molecules [4,19,20].

Recently, an HD TGC regimen has been successfully used in combination with other active antimicrobials in 22 critically ill patients with *K. pneumoniae* Carbapenemase-producing (KPC) *K. pneumoniae* infections (mainly VAP and BSI) [21]. The authors reported a high percentage of favorable response (88% clinical cure and 92% survival), probably due to the specific case mix of that population, represented by young trauma patients without significant comorbidities and septic shock.

TGC effectively penetrates skin, soft tissues and intra-abdominal organs but several concerns have been raised about its distribution in the lung. In a phase-3 trial [22] this molecule was compared with imipenem for the treatment of hospital-acquired pneumonia, without reaching non-inferiority criteria in the subset of patients with VAP. Moreover, some authors [23] have observed that TGC lung concentrations in intracellular epithelial lining fluid (ELF) were remarkably higher than in the extracellular fluid and serum. This observation has suggested the use of higher dosages in order to treat pneumonia caused by MDR pathogens. These data were also confirmed by a recent multicenter PK study in children affected by serious infections, where a dose of 1.2 mg/kg every 12 hours better correlated with the target AUC_0–24_:MIC_90_ ratios [24]. Hence, although TGC lung levels in infected patients are expected to be higher than healthy volunteers, standard doses are probably inadequate to reach maximally efficacy, especially against MDR pathogens on the upper end of the MIC distribution (1 to 2 mcg/ml). Recently, in a randomized phase-2 trial, treatment with HD TGC (100 mg every 12 hours) was associated with a better clinical response than imipenem treated patients, in nosocomial pneumonia caused by Gram-positive and Gram-negative bacteria. This clinical result was supported by the favourable PK-PD profile observed in HD TGC treated patients [6].

Few other authors have reported the treatment of MDR severe infections with TGC at higher than standard doses. Cunha [25] described the successful use of this molecule, up to 200 mg every 24 hours, during urinary tract infection/urosepsis, reporting no adverse effects. Similarly *Lewinski et al.*[26] reported a case of PDR *K. pneumoniae* pneumonia and bacteremia resolved with a combination of colistin and HD TGC. In general, TGC use is associated with few significant AE other than nausea and vomiting [27,28], but two recent meta-analyses documented that this molecule administration was associated with more AE than comparative drugs [29,30]. Not surprisingly, the most frequently reported AE (nausea and vomiting) had no impact in our cohort of critically ill patients, who were sedated and mechanically ventilated. To date there are limited data for AE related to the use of HD TGC [25,28]. In our cohort, the percentage of patients who manifested biochemical abnormalities, grading the AE, varied between 10% and 20%. Anyway, the retrospective design of this study does not allow an accurate estimation of the TGC contribution to these abnormalities. Hence, due to the high severity of our study patients, other factors might have exerted a crucial role affecting organ functions.

The overall ICU mortality observed in the entire cohort (100 patients) was high (57%) and only about half of patients achieved clinical cure or microbiological eradication (48% and 41%, respectively). These data might be explained by the relevant severity of illness: about 50% of them were in septic shock, 24% were receiving continuous renal replacement therapy (CRRT)and 35% had concomitant bacteremia. Recent reports suggest the usefulness of combination antimicrobial schemes including TGC, mainly during the treatment of KPC-producing *K. pneumoniae* infections [21,31]. However, despite the combined antimicrobial therapy strategy we have adopted, the percentage of IIAT was high (49%), and possibly responsible for the relevant rate of the observed unfavorable outcomes [32].

In our institution the use of HD TGC constantly increased over the study period, concomitantly with the shift towards less susceptible bacteria (Figure 2). The trend to increase TGC dosage used was probably influenced by the awareness of progressive increase in TGC MIC values in MDR germs isolated in our hospital, with the aim to avoid suboptimal doses for the management of difficult-to-treat infections.

Our study has several limitations. First, this is a single-center, retrospective analysis with a relatively small number of patients. Second, in almost all the patient TGC was used in addition to another active molecule and therefore we cannot draw any conclusion regarding the efficacy of HD TGC as monotherapy. Finally, we did not monitor the plasmatic and tissue concentrations that could confirm our clinical observations. However, to our knowledge, this is the largest comparative clinical study where the use of HD TGC has been described.

## Conclusions

These data suggest that TGC, used at doses higher than standard treatment, can be administered without relevant toxicity for the treatment of serious infections in critically ill sedated patients. The regimen with higher TGC doses (that is, 100 mg every 12 hours after a 200 mg loading dose) may be useful to improve the clinical outcome of patients with MDR Gram-negative VAP. Pharmacokinetic investigations and multicenter, prospective clinical trials are needed to confirm these preliminary results and investigate the efficacy of HD TGC in severe infections.

## Key messages

• The use of TGC at higher than standard doses is safe in critically ill patients

• The high TGC dosing regimen improves the outcome of patients with MDR Gram-negative VAP

## Abbreviations

AE: adverse events; AUC: area under the plasma concentration versus time; BSIs: bloodstream infections; cIAI: complicated intra-abdominal infections; CLSI: Clinical and Laboratory Standards Institute; cSSI: complicated skin and skin-structures infections; DHHS-CTCAE: Department of Health and Human Services - common terminology criteria for adverse events; ELF: epithelial lining fluid; FDA: Food and Drug Administration; HD: high dose; IIAT: initial inadequate antimicrobial treatment; KPC: *Klebsiella pneumoniae* Carbapenemase-producing; LD: loading dose; MALDI-TOF: matrix-assisted laser desorption ionization-time-of-flight; MDR: multidrug-resistant: MIC: minimal inhibitory concentration; MS: mass spectrometry; OR: odds ratio; SAPS II: simplified acute physiology score II; SD: standard dose; SOFA: sequential organ failure assessment; TGC: tigecycline; VAP: ventilator- associated pneumonia; XDR: extensively drug-resistant.

## Competing interests

The authors declare that they have no conflicts of interest.

## Authors’ contributions

GDP had full access to all the data in the study and takes responsibility for the integrity and the accuracy of the data analysis. GDP and MA conceived the study, and participated in its design and coordination and helped to draft the manuscript. LM and MT were in charge of the statistical analysis, participated in analysis and interpretation of data, helped to draft the manuscript, and critically revised the manuscript for important intellectual content. VB and GDP collected the data for the study and participated in statistical analysis. GB, VB, RM, MAP and TS participated in the conception, design and development of the database, helped in analysis and interpretation of data, helped in drafting of the manuscript and critically revised the manuscript for important intellectual content. All authors read and approved the final manuscript
